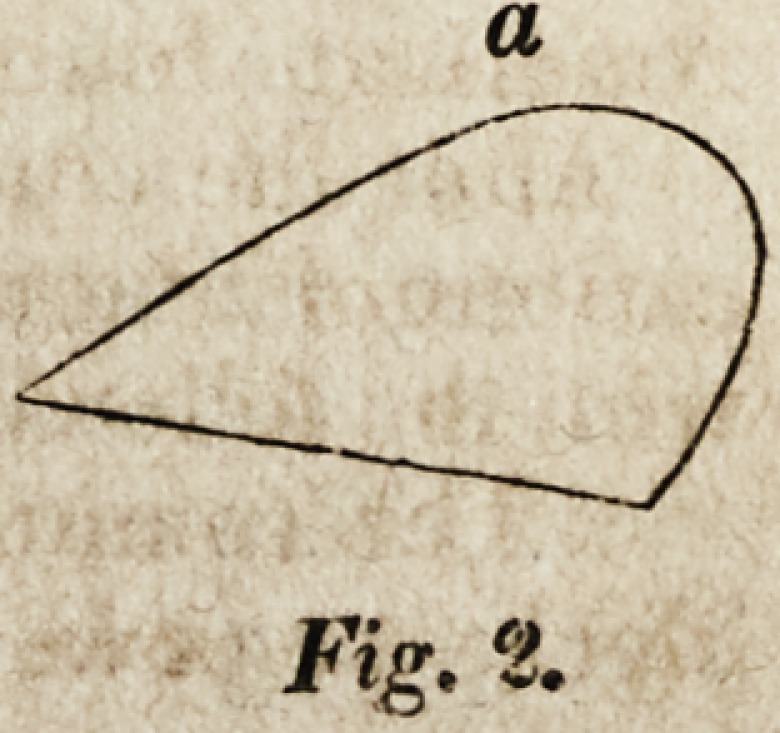# Surgery

**Published:** 1837-01

**Authors:** 


### SURGERY.

*On Neuralgias of the Urethra and Neck of the Bladder. From a Memoir read at the Academy of Sciences, July 6th,* 1835, *by* M. CIVIALE, D.M.P., Surgeon of l'Hôpital Necker, &c.

BY this name M. Civiale designates a very common affection of the genito-urinary organs, which is often attended with severe symptoms, but which cannot be explained by any discoverable organic changes. Hitherto, from the irregularity of its progress, the variability of the symptoms, the difficulty or impossibility of distinguishing it from other morbid states, it has been neglected by practitioners.

*Causes.* Rarely appreciable: it is generally observed in those whose nervous system has been over-excited by venereal excesses or moral emotions, or who have had neuralgia of other parts of the body. Prolonged irritation of the urethra and neck of the bladder, commencing stricture, constipation, diseases of the rectum and uterus, are circumstances favouring its production; but many are exempt from these causes.

*Symptoms.* Very vague and variable. The sensations, and the difficulty in making water, may depend equally on stone, organic disease of the prostate or bladder, paralysis of the bladder, stricture, &c. Notwithstanding this, the mobility of the symptoms points to their cause: they cease, reappear, increase, and diminish, without appreciable cause, and more rapidly than any other affection.

On these accounts, they do not fix the attention of the patient until their third or fourth return. At first, the frequent desire to make water, with difficulty and pain, comes on in paroxysms, with long intervals of perfect ease. To this is added an uncomfortable itching, with sensation of ardor, along the urethra, and particularly at the extremity of the penis; from whence it proceeds to the pubes, groins, sacrum, and kidneys. Urine almost always natural. These attacks rarely affect the general health. Sometimes they return at regular intervals; daily at the same hour, or every few days. The paroxysms generally increase in frequency and length as the disease continues. When they take on the quotidian type, they usually occur in the evening or night, or during digestion. Absence of occupation, all causes of excitability, and especially the fear of not being able to make water when needful, contribute to bring them on. The less water in the bladder, the greater difficulty and pain in voiding it. Fever very rarely accompanies the pain, however violent and obstinate, although the patient sensibly loses flesh. In process of time an organic disease is set up; and this nervous state is complicated with symptoms of catarrh, or some other severe alteration in the bladder or prostate. The rectum in time becomes affected, and the general health suffers. If the surgeon is consulted at this time, it is almost impossible for him immediately to recognize the primitive neuralgia, unless the patient gives (as he rarely does,) a clear history. If there is neither stricture, lesion of the prostate, nor stone in the bladder, the case is more simple; and, if there are complications, their severity is never in proportion to that of the symptoms. But local examinations are necessary to elucidate the case. Their effect is important in the diagnosis, for they ameliorate more often than they exasperate the disease. Inexperienced surgeons in these cases meet with difficulty in passing the bougie or sounds over the point which is the seat of the neuralgia; and this difficulty and pain are set down too easily as the effect of stricture. Such cases are more numerous than is imagined, and many modes of curing stricture owe their celebrity to these neuralgias. There are cases where instruments cannot be borne, in which profound lesions of the prostate and bladder are accompanied with excessive irritability of the urethra. Which was the primary affection can rarely be discovered. If a calculus is the cause, there is some hope; but, when there is chronic catarrh, with deep-seated alteration of the walls of the bladder or of its neck, carcinomatous tumours, &c., art is insufficient, even if a catheter could be introduced and retained.

*Treatment.* At their commencement these neuralgias are not serious. M. Civiale was led to a mode of treatment both easy and almost always successful, by the effects of the instruments of lithotrity. It would be imagined, when a patient about to be operated on had symptoms of excessive irritation, that the manipulations would aggravate his sufferings: more than once, however, M. Civiale has seen them diminished, and even cease. His indications are,—1. To diminish, by the contact of a foreign body, the increased and disordered sensibility of the urethra. 2. To produce a temporary disturbance of the function, and to break, by strong sensations, an inveterate habit of suffering. 3. To displace the irritation.

Sometimes, after accomplishing the first indication, the symptoms cease. M. Civiale has applied the well-known principle, that a bougie introduced, and kept some time in the urethra, renders it insensible. With this view he passes a soft bougie, of middle size, and leaves it for five or ten minutes; then withdraws it, and reintroduces it daily until it passes without pain; finally, he changes it for a larger one, if necessary. Ten or twelve introductions daily or every two days, according to the irritability of the patient, are generally sufficient in old cases, and fewer in recent ones. In all cases the symptoms diminished progressively. The bougie should not be suspended until it gives no pain. Towards the end, bougies of two and a half to three lines in diameter should be used, as they detect the commencement of strictures. If this does not succeed, a stronger impression must be made by the friction attending the introduction of a catheter, or of a lithotrity instrument. M. Civiale has seen a great many patients in which this plan has been pursued at the beginning, for the purpose of ascertaining if a stone existed, who were cured of the neuralgia by the shock alone. If the disease is still obstinate, and especially if it is accompanied with atony of the bladder, injections of pure water, of which the temperature is gradually diminished, contribute much to the cure. Irrigations are still more beneficial, even in cases apparently desperate. The last resource is revulsives, to displace the disease; such as purgatives, cold douche baths, or tartarized antimonial ointment to the hypogastrium and perineum. Other means should not be neglected. The state of the bowels is one of the most important, and M. Civiale has cured several by relieving obstinate constipation. The urine, if scanty and high coloured, should be rendered less acrid by baths, lavements, drinks, diuretics, alkalies, mild diet, &c. Such are the means which should generally be employed; but, in inveterate cases, the application of caustic, forcible injections, ligature of the penis near the glans (to prevent the escape of the urine), have been beneficial, by producing a stronger impression. They should not be employed unless a milder plan has failed. M. Civiale has found in such cases the light application of caustic over a considerable surface more particularly beneficial. In these inveterate cases the means should be varied, and persisted in for a length of time. Sometimes no treatment is beneficial; the amelioration is temporary and imperfect; the urethra does not become accustomed to the bougie. Repeated purgatives, cauteries, and even setons near the diseased spot, should be tried. M. Civiale has seen patients so discouraged as to leave off all treatment, and recover when they least expected it, and when they were doing nothing for it. Neuralgia should also be considered, in reference to treatment, as a complication of organic diseases of the genito-urinary organs. Thus, in catarrh of the bladder, which is often complicated with spasm or neuralgia of the urethra, the cure of the neuralgia sometimes modifies and diminishes the catarrhal affection, and favours the action of remedies. In organic strictures of the urethra, by removing the spasm and neuralgia, the symptoms are mitigated, and the chance of retention of urine prevented. In the treatment of stone, by removing the neuralgia, the operation is rendered possible, and even easily borne, which, from the nervous irritability of the neck of the bladder and urethra, was previously considered as likely to be very painful or impracticable. The neuralgias attending profound organic changes of the neck of the bladder are beyond the resources of art. *Gazette Médicale,* 18 *Juillet,* 1836.

---

### *On the Section of the Tendo Achillis in the Treatment of Club-feet.* By M. BOUVIER.

THIS is extracted from a memoir read before the Academy of Sciences, on the 5th and 12th of September, 1836. The variety of club-foot which consists of a forced extension of the foot is an effect of the permanent contraction of its extensor muscles and their tendon. The club-foot turned inwards is owing in a great part to the same cause. In both cases, the treatment consists in elongating the extensors of the foot by slow and sustained extension; the same end may be obtained by dividing the tendo Achillis, and retaining the ends apart: this is principally applicable in old cases, where machines only are often inefficient or dangerous, or where rapidity is essential. It was performed first in 1784, by Thilenius, near Francfort; since by Sartorius, Michaelis, and Delpech; and recently by Stromeyer. M. Bouvier has adopted a more simple operation than Stromeyer: he introduces under the skin covering the tendon a kind of needle, cutting on one side, by means of which he cuts the tendon through from its cutaneous surface inwards. The external wound is slight, and heals in a day or two. In a few days the foot is brought into its natural position, and the tendon unites in a few weeks without any signs of inflammation. Delpech and Stromeyer waited the commencement of union until they changed the wrong position of the foot; but M. Bouvier prefers separating the divided ends of the tendon immediately after the section: this prevents the pain caused by stretching the cicatrix, and does not risk the accident which once happened to Stromeyer, who found that the cicatrix could not be extended. M. Bouvier has traced in dogs the mode of reparation, and finds that a new portion of tendon is formed by successive transformations of the cellular sheath of the tendon. He illustrates his memoir with four cases.

The first case was that of a girl, æt. 14, who had had a club-foot since two years of age, owing to a scrofulous abscess. The section was made on the 15th of January last: at the end of a fortnight, the foot, which before the operation was in a line with the axis of the limb, formed with it nearly a right angle, and eight days after, it had passed this angle. The disposition of the tarsal bones, altered by the long continuance of the deformity, was the only delay of the re-establishment of the functions of the limb.

The second case was still more remarkable, on account of the advanced age and indocility of the patient, who would never submit to the proper application of the apparatus after the operation. The patient was a man forty-six years old, who had had a club-foot of the right side since six years of age. The external wound healed the second day. Three weeks after the operation, the foot formed a right angle with the leg, and the continuity of the tendon was re-established. He left the hospital at the expiration of forty days. He now walks on his flat heel, and makes long journeys on foot.

The third case was that of a girl, aged thirteen, who had had a club-foot from the age of four, in consequence of paralysis of the right side of her body. The exterior wound, the size of a leech-bite, healed on the day after the section was made. Immediately afterwards the foot was bent, and eight days after it formed a right angle with the leg.

The fourth case occurred in the practice of M. Roux. A boy, twelve years old, had retraction of the heel, and subsequently club-foot, from a wound in the calf when two years and a half old. The tendon was divided by M. Roux on the 4th of August, and on the following morning the foot was brought to a right angle. In three weeks the cicatrix of the tendon was firm, and there was no trace of the deformity.

M. Bouvier has at present two cases under his care, in both of which he has divided the tendon: one is a young man, æt. 23; the other a woman, aged 53, in whose case machines are also necessary. M. Bouvier presented (besides the casts of these cases,) the cast of the feet of the first patient, operated on by Delpech twenty years ago, who was accidentally met with in Paris. There has been no relapse, the cure being still perfect.

*Journal Hebdomadaire des Sc. Méd.* No. 38, 17 *Septembre*, 1836.

---

### *On the Radical Cure of Hernia in America.*

We have more than once taken a favorable notice of the invention by Mr. Stagner, of Kentucky, of a wooden headed truss, for the *radical* cure of hernia. Mr. S. is not a professional man, but his suggestion soon attracted the favorable notice of the medical gentlemen of the region in which he resided, and among the rest of Dr. Hood, a graduate, we believe, of Transylvania University; who visited the eastern cities, and sought to attract to the invention, the notice both of the people and the profession, and considerable success attended his efforts. It was soon discovered, however, that the form of Stagner's wooden pad (which was invariable) did not answer for every case; and this led to efforts, by different surgeons, to effect such modifications of figure, as might render it of general applicability. Among those who have exerted themselves in this laudable object, are Dr. Hood, whom we have just named, and Mr. Chase, of Philadelphia; each of whom has produced modifications of size and form, calculated, or at least intended, to augment its advantages. The deep interest of the subject induced the Philadelphia Medical Society to appoint a committee, "To investigate the character of Stagner's Truss, and other proposed means of radical cure in hernia;" and on the 5th and 12th of December, 1835, the committee, consisting of Drs. Reynell Coates, William Ashmead, and Isaac Parrish made their report, which was published in the American Journal, and from which we propose to give a few extracts.

After the citation of a number of cases, going to show the efficacy of the modifications of Stagner's block in restraining and curing Hernia, the report presents us with the following testimony as to the former point:

"The committee are decided in the opinion, that the retentive power of solid blocks exceeds, *cæteris paribus*, by considerable difference, that of pads composed of softer materials. If there could be any exception taken to this rule, it would be in favour of pads formed of very firm but highly elastic materials; but the only substance of the latter character, now employed in the construction of truss-pads, is the gum elastic, and against the direct application of caoutchouc to the skin there are strong physiological objections. Moreover, the committee is by no means prepared to advocate the superiority of elastic pads, in the present state of their knowledge. Whatever they may gain in facility of application, they lose in certainty of action. The great excellence of the solid truss-block is its perfect precision, and if required to adapt itself to changes of position in the part to which it is applied, it can be enabled so to do by the elasticity of the spring of the truss.

"Two circumstances should be stated in this place: the incompressibility of very firm pads and hard blocks renders it of the utmost importance that their form should be accurately adapted to the particular parts on which they are designed to act, and that they should be carefully placed and secured in correct relation with those parts. Carelessness in either of these respects would incur dangers, grave in just proportion to the power and usefulness of the apparatus. Hence considerable anatomical and surgical skill are requisite, both in the contrivance and the application of trusses armed with such pads or blocks, and they can never be permitted to pass, with safety, out of the hands of surgical scholars and practitioners. Again, the application of such machines, in early infancy, is deemed by the committee both unnecessary and improper.

"With regard to other dangers and inconveniences, your committee will merely remark, that the charge of danger of general peritoneal inflammation from the communicated irritation of the blocks, when their application is directed by competent skill, is deemed nugatory, and in opposition to both well-known pathological laws and the direct evidence of experience. The committee, therefore, think that these alleged dangers do not appreciably affect the permanent retentive power of the apparatus.

"There is apparently one slight additional security against the descent of the bowel in inguinal and certain other hernia, which is often consecutive upon the use of the hard block for a moderate length of time. This is the rapid absorption of the deposits in the subcutaneous cellular tissue, and sometimes in the dermoid tissue also, which permits the block to act almost immediately upon the tendinous canal, thus effectually closing the neck of the hernial sac, of which it very probably produces the obliteration. It is possible that the temporary security produced by this means, which very slightly opposes the formation of a new sac, has been a cause of deception with regard to certain cases reported as radical cures, but which have been subjected to relapse.

"In regard to the retentive power of particular blocks, the committee are prepared to express their warm approval of the inguinal block of Mr. Chase, [Fig. 1,] in which, at present, it can suggest no improvement. It also approves of Dr. Hood's ventro-inguinal block, [Fig. 2,] with the parabolic projection, but considers a more perfect instrument, to fulfil the same purposes, both possible and desirable."*

On the very important question of a *radical* cure, from the use of the wooden block, the report holds the following language:

"With respect to the question of the radical cure of hernia, by means of the best of the instruments which have passed in review, it should be borne in mind, that success in cases of umbilical hernia in young children is almost general, when methodical bandaging has been judiciously employed. That in other varieties of hernia, affecting subjects of similar ages, success is by no means rare, under the operation of trusses with soft pads; that in children over ten years of age, it becomes

*Fig.* 1.

* These figures give a section of the pads at the point of their greatest diameter; *a* being the side applied to the body. The sections are taken from the original Report in "The American Journal of the Medical Sciences."

*Fig.* 2.

rather uncommon; that in youths between the age of puberty and that of twenty years, it becomes rare; and after the latter period, *very rare.* These remarks premised, the committee are of opinion, that the chances of radical cure depend mainly upon the retentive power of the apparatus employed, and their opinion on this subject has been already expressed. It would be wrong to enter upon the calculation of probabilities, without much more extended observation than has yet been possible, but the committee have no hesitation in stating that the action of the several blocks recommended appears to offer much more prospect of radical cure, even under unfavorable circumstances, than any apparatus previously offered to the public, and which has fallen under their notice after considerable research."

As to the *modus operandi* of the block, in effecting a radical cure, the committee incline to the opinion, that an accurate, perfect, and constant retention of the protruding part, is far more important than the excitation of inflammatory action. We shall not follow them in their reasonings on this subject, which embrace a reference to the spontaneous closure of the *tunica vaginalis testis*, in childhood; for no certainty will attend our knowledge on this subject, until accumulated dissections shall have been made of those who have been radically cured; and of course this will require the lapse of many years.

*Western Journal of the Medical and Physical Sciences.* No. 35. 1836.

---

*Cases of Foreign Bodies introduced into the Stomach and Anus.*
By J. P. Dor. (*Thesis.*)

Case i. A man, æt. thirty-two, who had suffered in his bowels for some time, had nausea and vomiting, and passed, after much exertion, some liquid matters, slightly coloured by the rectum. There was some fulness and also dulness of sound in the middle of the abdomen, and these symptoms were replaced by the appearance of a middle-sized tumour, uneven, lumpy, and moveable under the hand. Disease of the mesenteric glands was suspected: two months after the commencement of the disease he died of peritonitis. On dissection, besides the marks of peritoneal inflammation, there was found considerable dilatation of the small intestine, with decided contraction of the large intestines. At the junction of the ileum with the cæcum, there was a sac with thick walls, adherent to the neighbouring parts, of the size of the head of a fœtus, and formed by the ileum. This sac contained 120 plum stones, some cherry stones, and ninety-two balls of lead, whose surfaces were completely indented with small facets. The ileo-cæcal valve, which corresponded to the lower part of the right side of this sac, was almost entirely obliterated, only leaving a small hole through which liquid might pass. All the other organs were healthy. From the history of the case, it was probable that the primary affection was a partial contraction of the intestinal cavity, which, hindering defecation, induced the patient to swallow at first fruit stones, and then balls, to overcome the obstacle which he supposed to exist.

Case ii. M. A. Berard, in his essay on Diagnosis, relates a somewhat similar case. An old woman had a large tumour at the umbilicus, giving an unequivocal sensation of crepitation. On dissection, considerable contraction of the intestine was found, behind which were accumulated more than six hundred cherry stones, and in front of this was a collection of pus. The sensation produced by the stones rubbing against each other was so similar to the crepitation of emphysema, that error was almost unavoidable.

The following three cases of foreign bodies introduced into the rectum happened at Marseilles. In the first, a wooden pestle was introduced by the small end, and the large end was deeply buried in the anus, so that neither forceps nor the fingers could have taken hold of it. Pincers jointed like the branches of the forceps were insinuated to some depth over the body of the pestle, and it was extracted. The patient, notwithstanding, died thirty hours afterwards, from inflammation of the rectum, the consequence of the attempts made by himself and the surgeon at extraction. The next case is one in which a young man introduced a fork into the anus. The prongs were situated too inches within the cutaneous margin of the anus, and penetrated the sides of the gut on the slightest traction. By means of forceps they were so compressed together as to form but one, and the extraction was then easy. The third instance is that of a young man who introduced into the rectum an eau de cologne bottle, which the efforts of defecation could not expel. M. Raymonet tried fruitlessly to withdraw it by means of the fingers and the usual instruments. It then struck him that he might remove it by introducing into the bottle itself (the neck being downwards) a pair of forceps whose branches were made to separate by a sliding ring, and as the neck of the bottle was sufficiently strong this plan answered. *Archives Gen. de Med. Juillet*, 1836.

---

*Statistical Results of Amputations.* By M. A. GENDRON. (*Thesis.*)

FROM the result of sixty amputations in adults, M. Gendron has drawn the following corollaries:—1. The chances of success are greater in proportion as the individuals have been for a long time exposed to a debilitating cause. Of twenty-four operations under such circumstances, there were fifteen cures, and nine deaths. 2. The chances are equal in patients who have been for a short time exposed to a debilitating cause, that is to say, in those in whom the disease, when they entered the hospital, has not required amputation, but in whom it has been obliged to be resorted to from subsequent accidents. Of eleven operations, there were four cures, and seven deaths. 3. The chances of success are very slight in amputations in vigorous individuals, immediately after accidents requiring the operation. Of eight operations, there were seven deaths, and one cure. 4. Union by the first intention, or immediate union, is in general preferable to mediate union or by granulation. Of sixteen cases of amputation, in which this latter was attempted, six were cured, and ten died: of the latter there were eight with unequivocal appearances, on dissection, of reabsorption of pus; of these eight, there were three who died at most two days after amputation, which ought not to be counted: there remain, therefore, five cases in which both the symptoms and the lesions of reabsorption of pus only appeared after the operation. Of the two fatal cases, which complete the original number of ten, the one died thirty-six hours after the operation, and the other between the tenth and eleventh day, which is the only one which can be counted, if the facts are examined with regard to reabsorption. Therefore, of six individuals in all who had no symptoms of reabsorption of pus before amputation, there was only one in which, on examination, traces of this disease were not found. It may, therefore, be concluded that, if union by granulation is not the cause of these secondary abscesses, it at least does not oppose their formation. Of twenty-nine amputations, where union by the first intention was employed, fifteen died, and fourteen were cured. All the bodies were not examined, and notwithstanding, even supposing that all those who were not examined after death had died from reabsorption of pus, the proportion of this kind of lesion would be much less than in the other series of cases.

[We have extracted these particulars, not for the value of the corollaries as universally applicable, but from their affording some means of forming a judgment on the relative success of amputations in France and England, and from their furnishing data towards estimating the advantages of the two very opposite modes of treatment most prevalent in the two countries; that of simply bringing the edges of the skin together, and lightly dressing the wound so as to produce union with the first intention, (which is the almost universal custom here,) or of stuffing as much charpie as possible into the wound for the purpose (which it fully answers,) of producing suppuration, as is the most usual plan in the Paris hospitals.]

*Archives Gen. de Med. Juillet*, 1836.

---

*On Inguino-interstitial Hernia.* By G. GOYRAND, D.M.

THIS name is applied to the form of hernia termed by most authors *incomplete inguinal*, and described by Boyer as *intra-inguinal*. The author objects to the former term; because, whatever may be their situation, when the viscera have escaped from the abdomen, the hernia is complete; and to the latter, because the inguinal canal does not always constitute the limits of the protruded viscera.

M. Goyrand has had several opportunities of examining the nature of this form of hernia. Its seat is the inguinal canal, but, if voluminous, it extends outwards, towards the iliac spines, separating the inferior layers of the lesser oblique, and even of the transverse muscles, from the fascia transversalis. It has two membranous coverings,—the sac and the serous investment of the spermatic cord. The cavity in which it is contained is bounded, anteriorly, by the aponeuroses of the great oblique muscle, and by the inferior fibres of the lesser oblique, and the origin of the cremaster; posteriorly, by the fascia transversalis; above, by the inferior border of the transverse, and some fibres of the lesser oblique muscles. The aperture by which it escapes from the abdomen is generally the opening in the fascia transversalis, through which the spermatic cord passes; on the internal side of which are the epigastric vessels, and inferiorly the spermatic cord. Superiorly and externally, the hernia is in connexion with no vessels of importance. The inguinal ring forms the inferior orifice of this cavity. The neck of the sac is embraced by the abdominal opening, and these parts are adherent in an old hernia. The hernia extends more easily through the inguinal canal than towards the iliac spines; since, in the latter direction, it must make a passage between the muscular fibres. Thus, the communication of the accidental cavity with that of the abdomen is commonly situated nearer to the external than to the internal extremity of the swelling. A portion of the hernia may project beyond the inguinal ring, when it will appear to consist of two distinct portions. It may be congenital, and the testicle is then situated on the inferior wall of the hernial cavity, and projects at the posterior and inferior part of the sac. Its coverings, in the order of their position, from without inwards, are, the skin, adipose tissue and fascia superficialis, the aponeuroses of the external oblique, sometimes in old hernia very thin; a muscular layer, thin and pale, formed by the fibres of the internal oblique and cremaster, which also in old ruptures is scarcely recognizable; the cellular prolongation of the fascia transversalis and the sac. Between the last two there may be a degeneration of a fibrous or fatty nature, of the subserous cellular tissue. The inguino-interstitial hernia does not always escape from the internal inguinal aperture: its point of origin may also be external to this aperture, and consequently external to the epigastric artery, or it may be within, and having the epigastric artery on its outer side.

The local symptoms of this hernia are, an ovoid, flattened tumour, situated obliquely in a line, drawn to the pubes, from the anterior and superior spine of the ilium. But, if the tumour be small, if the fascia of the external oblique should be resistant, and of its normal structure, or the patient fat, the diagnosis becomes difficult. In consequence of the resistance of the fascia, in one of the cases which is related, although six inches of intestine were excluded, and the patient was not very fat, there was no appreciable swelling. Before dividing the stricture, in the operation, the aperture at which the viscera have escaped from the abdomen may be ascertained by following the spermatic cord towards the internal inguinal opening. The author recommends several small incisions to be made in the stricture, in preference to one large incision; as there is, in consequence, less danger of hemorrhage, and less subsequent weakness of the abdominal parietes at that point.—*Mémoires de l'Academie Royale de Médecine*, tome v. 1836.

---

*Remarks on some of the remote Effects of Fractures of the Femur.*
By Dr. J. Guyot.

The object of this paper is to notice an affection of the femur, occurring subsequent to its fracture, at a period more or less remote from the time of consolidation. The author has found it no where noticed by surgical writers, and he is further disposed to believe that it has not been recognized, as the cases are generally considered as without the sphere of surgical treatment. An analysis of the first case will present the principal points of importance.

M. Turgot, in consequence of a fall from his horse, had suffered a fracture of the femur, at the junction of its upper with its middle third. This had happened three years and a half ago. At the time of its occurrence M. Dupuytren had treated the patient; but, in consequence of a change which was made in the apparatus, during the process of union, consolidation had been very slow, and was followed by deformity. The patient was able to walk with the assistance of a short crutch. Subsequently, the callus became swollen and excessively painful; the whole limb œdematous, and of a violet colour. The limb was unable to support the body, and the least contact of the foot with the ground gave rise to extreme pain. From this time onwards, for three years, no means were efficacious in restoring the callus to its original state. After the use of treatment directed to the general health, there was some amelioration; but the patient was obliged to support the limb, and very trifling causes of irritation sufficed to bring on an inflammatory condition of the callus, accompanied with pain, swelling, increased heat, and spasmodic actions of the muscles. This condition, accompanied with much constitutional disturbance, lasted on one occasion for about nine days. On examining the limb, M. Guyot found that there was an overlapping of the broken extremities of the bone, with an enormous deposition of callus; the neighbouring parts being much engorged. After having in vain employed various mechanical contrivances, M. Guyot confined his patient entirely to bed, and applied the apparatus of Boyer, without any compresses, the action of which was simply to extend the limb by its two extremities, like the string of a bow. In two or three days' time, it was necessary to employ a mechanical bed. Eight days afterwards, the limb had acquired its natural size and colour: the extending force was slightly augmented every day. In consequence of a slight effort made by the patient, there was return of pain in the same parts. A large blister was applied, and several times repeated, at intervals of a few days. Six weeks after the commencement of this treatment, M. Turgot was able to support himself on the diseased limb, and, after a large cauterization of the thigh, posterior to and on a level with the situation of the fracture, the symptoms were gradually and entirely removed, and the patient was able to walk without the assistance of any support.

The second case which is related is not complete. In the third, the same class of symptoms occurred six months subsequent to a fracture of the cervix femoris, and continued, with various degrees of intensity, until M. Guyot was consulted twelve months after the accident. The application of blisters, and subsequently of cauterization, were effectual in this case to restore the limb to its functions.

M. Guyot considers that the nature of the affection is an inflammation of the callus, of a chronic character, situated in its interior, and extending to the periosteum and fibrous textures connected with it; and that counter-irritants, such as blisters and cauteries, are the only effectual means of treatment.

*Archives Génerales de Médecine. Fevrier*, 1836.

---

### *On Inflammation of the Superficial Mucous Glands of the Male Urethra.* By Dr. W. KLEEBERG, of Königsberg.

Dr. KLEEBERG looks upon this affection of the urethra as similar to the disease of the mucous follicles of the genital organs of the female, described by Dr. Fricke, of Hamburg. He thinks that it is sometimes combined with clap or gleet, and sometimes not; and that it can be distinguished from them by an accurate examination of the orifice of the urethra, and by its not being amenable to the usual anti-gonorrhœal remedies. The disease attacks the outermost row of mucous follicles, which lie close under the tender skin of the glans, and whose orifices are distinguished with difficulty in the healthy state. The inflammatory affection of these follicles may arise as an idiopathic disease, but it is most frequently associated with gonorrhœa: it generally exhibits in its course the following phenomena:—The immediate circumference of the mouth of the urethra becomes swollen, and of a brownish red colour; and the pain, which is but slight, is scarcely increased during the passing of water. In consequence of the inflammatory swelling, the mouths of the glands become obstructed, and, in the course of two or three days, pustules are formed in their places, which break and discharge a yellowish pus. The orifices of the large mucous follicles are now seen dilated and surrounded by a swollen dark-red border, and they discharge a muco-purulent fluid into the urethra; if this be washed off, and the glans compressed, the fluid is distinctly seen issuing from these openings. Besides this blenorrhœa of the internal walls of the mucous follicles, there may be also suppuration established round the external walls, as the result of a more intense degree of inflammation; and, in this case, larger pustules are observed at some distance from the mouth of the urethra, which burst, and leave behind an excoriated surface, instead of an inflamed follicle. Occasionally, the disease spreads more deeply into the urethra, and after the lapse of two or three weeks, during which the patient had not exposed himself to any fresh infection, he observes, to his astonishment, the sudden appearance of a gonorrhœa, which he is inclined to attribute to the surgeon's ignorance of the first symptoms of his disease. The dot-shaped openings observable near the orifice of the urethra in this disease look like minute chancres, and are sometimes mistaken for such. Sympathetic buboes also appear; but they never, as in case of gonorrhœa, attain a remarkable degree of inflammation. The enlargement of the glandular orifices does not disappear for several weeks after the disease is cured.

The chronic stage of this affection is distinguished by a permanent copper-coloured blush round the orifice of the urethra. Besides this, there is scarcely any other symptom of disease, except a slight watery secretion, which glues together the lips of the urethra at night. By holding up the orifice of the urethra and pressing the glans, minute whitish flakes are seen to issue from the mouths of the mucous follicles.

The treatment of this affection always requires the employment of appropriate local applications, in addition to the remedies prescribed for gonorrhœa, whether it be accompanied by the latter or exist as an idiopathic disease. In the acute stage, Dr. Kleeberg uses the saturnine lotion, and afterwards a weak solution of corrosive sublimate applied on charpie. From his own experience, he is convinced of the advantage of opening the pustules at an early period, to prevent the inflammatory swelling and obstruction of the glans. In the chronic stage, he employs solutions of corrosive sublimate or caustic potash. In cases of obstinate blenorrhœa from the cavities of the glans, he has found the introduction of a piece of caustic scraped to a fine point the only efficient means of cure.

*Zeitschrift für die gesammte Medicin,* Band ii. Heft 2.

---

*On the Elevation and Depression of the Pelvis, in Luxations of the Femur; and on certain forms of Lameness, hitherto undescribed.* By Jules Guerin, M.D.

In dislocations of the femur upwards and outwards, the pelvis is always elevated on the luxated side, in a degree proportionate to the surface of the ilium, over which the head of the bone has passed. The fact, which is constant, is thus explained:—In passing upwards on the external surface of the ilium, the superior extremity of the femur drags with it the united tendons of the psoas and iliacus muscles, which are inserted into the lesser trochanter. These tendons, pressing against the inferior part of the anterior border of the ilium, upon which they are reflected as in a pulley, elevate the pelvis, because they are unable to accommodate themselves to the distance which would exist between their two points of insertion, supposing the pelvis to remain fixed. The part where this pressure is effected, by the united muscles, is shown by a depression, more or less deep, at the base of the anterior inferior spine of the ilium. The principal consequences of this fact are the following:—1. In all the luxations of the femur upwards and outwards, the shortening of the luxated limb is greatly owing to the elevation of the pelvis. 2. The more complete the luxation, the greater is the elevation of the pelvis. 3. In old double or congenital luxations, the bending of the loins and the elevation of the pelvis forwards, are the consequences of the double ascent of the inferior attachment of the psoas and iliacus on the external surface of the ilium. 4. In the other dislocations of the femur, the pelvis is always influenced by the relations between the origin and insertion of these muscles: thus, in the dislocation upwards and forwards, where the inferior insertion approaches the superior; the pelvis is elevated on the opposite side, and, by its depression on the luxated side, completely destroys the appearance of shortening of the affected limb.

The operation of the same cause is evident in the following circumstances:—5. In the disease of the hip-joint, where the elevation or depression of the pelvis gives the appearance of shortening or elongation to the limb; the action of the psoas and iliacus is the cause of the phenomena. In the early period of this disease, the pain which induces the patient to incline the trunk towards the diseased side relaxes the psoas, and causes a depression of the pelvis on that side; whilst, from an opposite cause, it is elevated on the other side. In the second period, when the bone is partially or completely dislocated, and there is frequently contraction of the psoas, all these causes combine to produce elevation of the pelvis on the diseased side. 6. After the reduction of the majority of old dislocations of the femur, and even after the cure of hip-disease, although the limbs are of equal length and the articular surfaces exactly in contact, there remains a lameness, which depends on the elevation of the pelvis. 7. There is a congenital or acquired lameness, in which there is an apparent shortening, although the two limbs are of exactly the same length, and the articular surfaces accurately in contact; and this species of lameness, hitherto unnoticed, is owing to an elevation of the pelvis, on the side which appears the shorter. *Gazette Medicale*, No. 15, 1836.

---

*On a new Mode of treating Herpes, (Dartres.)* By Dr. BUGLIARELLI.

THE success of the following treatment of herpetic eruption is confirmed by thirteen cases, in which a cure was speedily effected.

Five pounds of sublimated sulphur and eight of common oil are to be mixed in a glass matrass, with a large mouth, well luted. They are then to be gradually heated in a sand-bath, the heat being increased until the sulphur is quite liquefied. The mixture, when reduced to two pounds, is to be allowed to cool, and then, after five pounds of alcohol are added, it is to be again reduced to two pounds, by the same means; after which, on separating the residue, a spirituous oil is obtained which, when united to an equal quantity of muriatic acid, forms the antiherpetic liquor of the first degree. This is fitted only to the chronic form of the disease, and is to be used occasionally in the course of the treatment. One part of this liquid united with two parts of the distilled water of elder-flowers constitutes that of the second degree, which is useful in old indolent forms of the disease. The addition of three parts of elder-flower water to one part of the liquor of the first degree, constitutes that of the third degree, when it may be employed in recent cases and when the skin is very sensitive. Half a pound of the first liquor, mixed with a similar quantity of the second, is sufficient to cure an eruption which occupies the whole cutaneous surface, and that within two months.

This remedy is used internally as well as externally, its action being aided by other means. The author commences his treatment by purging with Epsom salts; on the third day he prescribes warm soft-water baths, diaphoretics, and Ethiops mineral. The warm bath is repeated twice a week, and in it are boiled one pound of sulphur and half a pound of quick lime. The diseased spots are touched daily, by means of a small brush, with the liquid.

If the patients would not submit to the use of the protosulphuret of mercury, the anti-herpetic liquor of the first degree was substituted; the dose being from ten to thirty drops in a pound of the diaphoretic decoction. The diet should be more vegetable than animal, and saline and acidulous substances should be avoided.

*Giornale delle Scienze Mediche, per la Sicilia.* 1835.

---

*New Method of reducing Luxations of the Humerus.* By M. C. GERARD. *(Thesis.)*

M. GERARD has employed the following plan in thirteen cases of dislocated humerus, during the last fifteen years. All the cases were recent; he thinks it advisable in all the kinds of dislocation. The patient being seated in a chair, an assistant, placed on the side opposite to the luxation, passes his arms around the neck of the patient, and, crossing his hands over the luxated shoulder, opposes the efforts made by the surgeon to replace the bone. The surgeon, stationed on the injured side, places his left forearm beneath the upper part of the dislocated bone, as near as possible to the armpit. He then approaches his patient so closely as to allow the cubital end of the dislocated humerus to rest against his own side, whilst he supports it longitudinally as near as possible to the trunk of the patient. The surgeon then draws the articulation in a direction upwards and outwards, and, without using much force, the bone is immediately replaced.

*Archives gén. de Méd. Juillet,* 1836.

---

*New Treatment of Strictures of the Urethra.* By M. Jobert, Surgeon to l'Hôpital St. Louis.

Having ascertained the situation of the stricture, M. Jobert oils a bougie, and then dips it into calcined alum reduced to an impalpable powder: if the obstacle is considerable, he dips the bougie again in oil, and afterwards in calcined alum, so that there are two layers of alum upon the bougie. He introduces the instrument gradually, and presses it softly against the obstacle, and fixes it in the urethra by four tapes. Sometimes two hours are sufficient to conquer the obstruction, and allow the patient to pass his water; at other times, it does not succeed until the next morning. It is necessary to introduce the bougie, similarly medicated, for many days successively, until it reaches the bladder. M. Jobert has found the most obstinate strictures yield to this treatment: the inflammation it produced was moderate, and the discharge soon ceased.

[We have had no experience of this plan.]

*Journal Hebdomadaire des Sc. Médicales,* 10 *Sept.* 1836.

---

*Obliteration of the Brachial Artery by compression of a Pad.* By J. P. Dor. (*Thesis.*)

A child, twelve years old, was admitted into the Hôtel Dieu for fracture of the clavicle, which was treated by Dessault's plan, in which a cushion is placed in the armpit as a fulcrum for the humerus. After the third application, he complained of pain in the inner part of the arm; but the bandages were not removed until the thirtieth day, when the fracture was consolidated, but there was an eschar at that part of the forearm which was the fixed point of the lever formed by the humerus upon the cushion, and the brachial artery was obliterated. Sensibility and heat of the limb were preserved, but it was very stiff. Some days afterwards, pulsations gradually returned in the radial artery, and were perfectly restored when the child left the hospital, on the eschar healing.

*Archives gén. de Méd., Juillet,* 1836.

---

*On Sulphuret of Lime in Diseases of the Skin.* By Dr. Savardan.

Dr. Savardan has employed the following ointment in chronic diseases of the skin, for the last twelve years, with very great success: eight parts of lard are intimately mixed with one part of sulphuret of lime; and one drachm is directed to be rubbed into the palms of the hands for one quarter of an hour night and morning. Dr. S. has given short notes of thirty cases of chronic diseases of the skin of various kinds affecting different parts of the body, all of which gave way to this ointment, used in the manner specified. All were cases of long continuance, and the treatment was of course protracted; one or two yielding in rather more than a month, others in three, four, seven months; whilst in others the frictions were persevered in from one to two years.

*Journal des Connaissances Médico-chirurgicales, Janvier,* 1836.

## Figures and Tables

**Fig. 1. f1:**
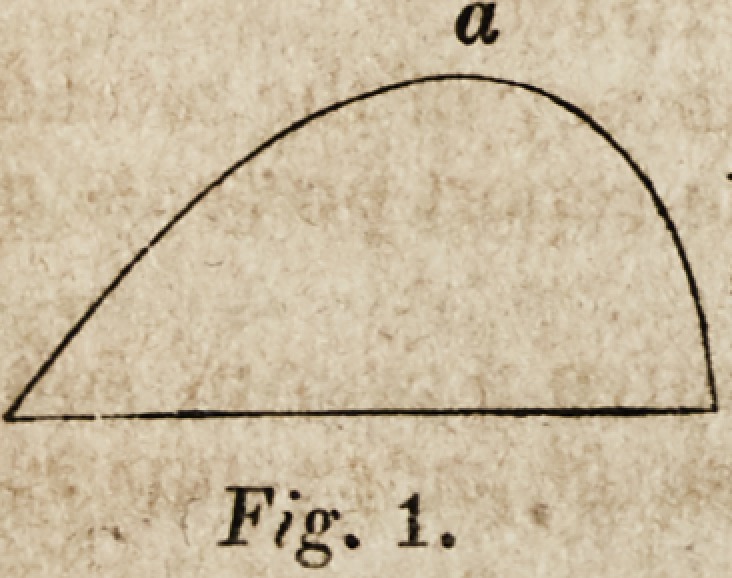


**Fig. 2. f2:**